# ASSOCIATION BETWEEN COGNITION AND FALL RISK BASED ON THE STEADI ALGORITHM: PROJECT VIBE

**DOI:** 10.1093/geroni/igz038.1068

**Published:** 2019-11-08

**Authors:** Tiffany F Hughes, Cara Carramusa, Daniel J Van Dussen

**Affiliations:** Youngstown State University, Youngstown, Ohio, United States

## Abstract

Falls are a growing concern among older adults with estimates that one in four fall each year. Older adults who experience a fall are at higher risk for poor health outcomes that threaten independence and increase risk of death. Impairment in cognitive function is known to be associated with greater fall occurrence; however, cognitive testing is not an integral part of clinical fall risk assessment. The purpose of this study is to examine cognitive performance in relation to fall risk level and its components determined using the Stopping Elderly Accidents, Deaths, and Injuries (STEADI) algorithm. One hundred eight community dwelling older adults (mean age 79(SD 7.3) years, 76% women, and 56% college or higher education) were included. Cognition was assessed with the Montreal Cognitive Assessment (MoCA; >= 26 normal). The STEADI algorithm was used to assess fall risk (low vs. moderate/high) based on the Stay Independent screening (>= 4), impairment in gait (Timed Up and Go (TUG)), strength (30-second chair stand), and balance (4-stage balance), and number of falls (>= 2). Associations between cognition and fall risk and its components were assessed using logistic regression adjusting for age, gender, and education. Normal cognitive status was marginally associated with lower likelihood of moderate/high compared to low fall risk (OR 0.42, 95% CI 0.17-1.04), and with a lower likelihood of TUG impairment (OR 0.22, 95% CI 0.07-0.66). These results suggest cognitive status may contribute important information about older adults’ fall risk and should be considered an integral part of fall risk assessment.

